# Development and characterization of topical formulation for maintenance therapy containing sorbitan monostearate with and without PEG‐100‐stearate

**DOI:** 10.1111/ics.13023

**Published:** 2024-09-16

**Authors:** Hans Schoenfelder, Yvonne Wiedemann, Dominique Jasmin Lunter

**Affiliations:** ^1^ Department of Pharmaceutical Technology, Faculty of Science Eberhard Karls Universität Tübingen Tuebingen Germany

**Keywords:** basic therapy, formulation/stability, skin barrier, skin physiology/structure, sorbitan ester, trans‐epidermal water loss

## Abstract

**Objective:**

Basic therapy is an integral part of the treatment of chronic skin diseases. However, the formulation of skin products should be analysed with respect to the physical stability and tolerance by the patients before applying them to diseased skin. In particular, the suitability of the formulation for use on damaged skin should be taken into consideration so that no exacerbation of the condition is caused.

**Methods:**

The following approach investigated two formulations with the emulsifier sorbitan monostearate and one with the addition of polyethylene glycol 100 stearyl ether. The characterization included rheology, macroscopic and microscopic cream analysis compared to marketed products for basic therapy. Pyranine staining of stratum corneum (SC) and trans‐epidermal water loss (TEWL) measurements were performed with ex vivo porcine SC to asses skin barrier function.

**Results:**

The rheological characterization showed a gel‐like, viscoelastic behaviour of the formulations and a viscosity in the same order of magnitude as the marketed products. Staining with pyranine revealed that skin damage caused by sodium lauryl sulfate was compensated by treatment with the developed formulations. Following the same trend, TEWL results clearly showed decreasing values, which evidence improved skin barrier function.

**Conclusion:**

In conclusion, the developed sorbitan monostearate formulations can potentially improve deficient skin barrier function as a part of basic therapy of skin diseases and act as a superior alternative to market products comprising a minimum of well‐chosen ingredients.

## Graphical abstract

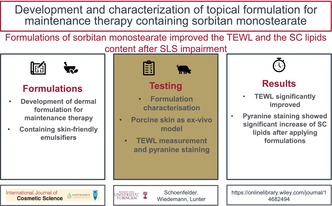

## Résumé

**Objectif:**

la thérapie de base fait partie intégrante du traitement des maladies chroniques de la peau. Cependant, la formulation des produits pour la peau doit être analysée en termes de stabilité physique et de tolérance par les patients avant de les appliquer sur la peau malade. En particulier, il convient de prendre en compte l'adéquation de la formulation à être utilisée sur une peau lésée afin d'éviter toute exacerbation de l'affection.

**Méthodes:**

l'approche suivante a étudié deux formulations avec l'émulsifiant monostéarate de sorbitane et une autre avec l'ajout d'éther de stéaryle de polyéthylène glycol 100. La caractérisation comprenait la rhéologie, l'analyse macroscopique et microscopique de la crème par rapport aux produits commercialisés pour la thérapie de base. Une coloration à la pyranine de la couche cornée et des mesures de la perte d'eau transépidermique ont été effectuées avec des couches cornées ex vivo de porc pour évaluer la fonction de barrière cutanée.

**Résultats:**

la caractérisation rhéologique a montré un comportement viscoélastique de type gel des formulations et une viscosité du même ordre de grandeur que les produits commercialisés. La coloration à la pyranine a révélé que les lésions cutanées causées par le laurylsulfate de sodium étaient compensées par le traitement avec les formulations développées. Suivant la même tendance, les résultats de la perte d'eau transépidermique ont clairement montré des valeurs en baisse, ce qui témoigne de l'amélioration de la fonction de barrière cutanée.

**Conclusion:**

en conclusion, les formulations de monostéarate de sorbitane développées peuvent potentiellement améliorer la fonction de barrière cutanée déficiente dans le cadre d'une thérapie de base des maladies de la peau et constituer une alternative supérieure.

## INTRODUCTION

The treatment of various chronic skin diseases is a significant challenge. The most common therapies combine basic therapy with API‐ (active pharmaceutical ingredient) containing formulations [1, 2, 3]. Basic therapy involves applying nourishing topical products with or without special additives to improve or assist the therapy of skin diseases [2]. Cosmetic products are commonly applied in various galenic forms, mostly creams or ointments. The composition of a formulation offers various possibilities but also poses grand challenges. Specifically, the used ingredients should be examined for their effects on skin. Emulsifiers like sodium lauryl sulfate (SLS) turned out to be skin‐disruptive [4, 5, 6, 7, 8]. Zhang, Liu, and Lunter characterized alternative emulsifiers for dermal application with respect to their potential to extract lipids from the skin and thus impair skin barrier function. Most of the investigated emulsifiers turned out to be non‐skin‐irritative. However, some emulsifiers, like C20 (Polyethylene glycol‐20 cetyl ether), did impair the skin barrier function [9, 10, 11, 12, 13]. The objective of the following study was to develop formulations for basic therapy that contain a minimum of selected ingredients, including non‐skin‐irritative emulsifiers to support the treatment as much as possible. Psoriasis and dermatitis could be a potential field of use. The hydrophilic formulations contain rather large amounts of the oil phase, which in turn contains a relevant amount of white soft paraffin, which may exert a semi‐occlusive effect on the skin; thus, the formulation will be suitable for rather dry skin. Two formulations were developed with the emulsifier sorbitan monostearate (HLB 5 due to Croda). One formulation contained only sorbitan monostearate as emulsifier while the second formulation, additionally, contained polyethylene glycol 100 stearyl ether (S100) (HLB 19 due to Croda). S100 was also analysed in the past and found to be skin‐friendly [14]. The skin thickness and lipid content, measured by confocal Raman microscopy, were not reduced in comparison to other PEGs. So it was concluded not to extract lipids from the skin. Linoleic acid and cholesterol were added in an equimolar ratio similar to their natural occurrence in skin to substitute for the decreased lipids content of diseased skin [9, 13]. Medium‐chain triglycerides (MCT) were used as oil phase and are also proven to improve skin health [15]. The combination of linoleic acid, cholesterol, and MCT can be seen as the components that improve skin barrier function. White soft paraffin was used to obtain a cream of medium viscosity. The formulations were then characterized by their rheological behaviour, macroscopic and microscopic appearance, physical stability and the ability to improve the skin barrier function. TEWL measurements were used to this end in combination with pyranine staining to visualize the lipids in the SC [9, 16].

## MATERIALS AND METHODS

### Materials

SLS, sodium chloride and potassium chloride were obtained from Caesar & Loretz GmbH (D‐Hilden, Germany). Di‐sodium‐hydrogen phosphate and potassium‐di‐hydrogen phosphate were obtained from Carl Roth GmbH & Co. KG (D‐Karlsruhe, Germany). MCT (medium‐chain triglycerides, Myritol® 312) were obtained from BASF (D‐Ludwigshafen, Germany). Sorbitan ester sorbitan monostearate (Span 60) and Brij S100 (Polyethylene glycol 100 stearyl ether) were purchased from Croda GmbH (D‐Nettetal, Germany). Methyl orange was obtained from Merck KGaA (D‐Darmstadt, Germany). Blue‐violet (Dragocolor® KSMFST) was obtained from Symrise AG (D‐Holzminden, Germany). Trypsin (from porcine pancreas, lyophilized powder, type II‐S) and trypsin inhibitor (from glycine max [soybean], lyophilized powder) were obtained from Sigma (Sigma‐Aldrich Chemie GmbH, D‐Steinheim, Germany). Linoleic acid was obtained from Thermo Scientific (Thermo Fisher Scientific GmbH, GB‐Heysham, Great Britain). Pyranine was obtained from Alfa Aesar (GB‐Heysham, Great Britain). White soft paraffin (Vaselinum album) was obtained from Hansen & Rosenthal GmbH & Co. KG (D‐Hamburg, Germany). Lipo Lotio Cordes® was obtained from Ichthyol‐Gesellschaft (Cordes, Hermanni & Co., GmbH & Co. KG, D‐Hamburg, Germany). CeraVe moisturizing creme was obtained from L'Oréal Deutschland GmbH (D‐Düsseldorf, Germany). Alfason® Repair was obtained from Karo Pharma AB (S‐Stockholm, Sweden). Parafilm® was obtained from Bemis Company Inc. (WI‐Oshkosh, USA). Ultra‐pure water was generated by Elga Maxima (GB‐High Wycombe, Great Britain). Phosphate‐buffered saline (PBS) was prepared using sodium chloride and potassium chloride obtained from Caesar & Loretz GmbH (D‐Hilden, Germany), di‐sodium‐hydrogen phosphate, and potassium‐di‐hydrogen phosphate obtained from Carl Roth GmbH & Co. KG (D‐Karlsruhe, Germany). Porcine ear skins (German landrace; age: 15–30 weeks; weight: 40–64 kg) were provided by a local butcher. The Department of Pharmaceutical Technology at the University of Tuebingen has been registered for the use of animal products (registration number: DE 08416105221).

### Preparation of formulations and solutions

The two different formulations were manufactured by a mixing system (Topitec Automatic, Wepa Apothekenbedarf GmbH & Co KG, D‐Hillscheid, Germany). The compositions are listed in Table 1. The oil phase was heated to 80°C and then cooled to room temperature under stirring. After combining the water and oil phases, both after cooling down to room temperature, they were mixed. As mixing parameters 2, 4 and 6 min at 1000, 1500 and 2000 rpm mixing speed were evaluated. The optimal configuration was set to 4 min and 1500 rpm.

**TABLE 1:** Overview of formulations (*F*) regarding composition. *R* stands for recipe and final version used for rheology, TEWL measurement, and staining.

	F1‐1	F1‐2/R1	F2‐1	F2‐2	F2‐3/R2
White soft paraffin	65%	40%	50%	50%	35%
Cholesterol	0.13%	0.13%	0.13%	0.13%	0.13%
Linoleic acid	0.085%	0.085%	0.085%	0.085%	0.085%
Span 60	5%	5%	1%	5%	5%
S100	–	–	1%	5%	5%
MCT 312	10%	10%	10%	10%	10%
Water	Ad 100%	Ad 100%	Ad 100%	Ad 100%	Ad 100%
Time	2 min	2 min	2 min	2 min	2 min
rpm	1000 rpm	1000 rpm	1000 rpm	1000 rpm	1000 rpm
Phasing	O/W	O/W	O/W	O/W	O/W

An SLS solution was used to impair the skin barrier. It consisted of 1% SLS (w/w) in an aqueous solution.

### Formulation characterization

Rheological measurements were carried out using a Physica MCR 501 rheometer (Anton Paar GmbH, AT‐Graz, Austria) with a plate/plate geometry (PP 25; diameter: 25 mm; gap size: 0.2 mm). The temperature was maintained at 32°C for all measurements to simulate skin temperature. After the samples were applied to the plate, the recovery and equilibrating time of 2 min was set. To determine the dynamic viscosity η, the shear stress was measured as a function of the shear rate in the 1–1000 s^−1^ range. An oscillatory amplitude sweep test was performed in the strain range of 1–1000% deformation and a frequency of 6.28 rad s^−1^ to analyse the viscoelastic behaviour. The linear viscoelastic (LVE) range was determined. Subsequently, the frequency sweep tests were conducted in the angular frequency (ω) range of 100–1 rad s^−1^ and a strain amplitude of 1% deformation. The viscoelastic characteristics were revealed by the storage modulus (G') and loss modulus (G"). All measurements were performed in triplicate [13].

The phasing of the creams was investigated using the water‐soluble dye methyl orange and the oil‐soluble dye blue‐violet to stain the water or oil phase, respectively.

### Optical microscopy

The formulations were characterized by a polarization microscope (Microscope Axio Imager Z1, Carl Zeiss AG, D‐Oberkochen, Germany) with a 20× objective (numerical aperture 0.8, Plan‐Apochromat, Carl Zeiss AG, D‐Oberkochen, Germany) and respective software Zeiss Zen Blue (Carl Zeiss AG, D‐Oberkochen, Germany). The formulations were examined for homogeneity and the presence of cholesterol needles [13].

### Porcine ear skin preparation

Porcine skin was chosen as a surrogate for human skin [17, 18] because the skin is histologically and morphologically comparable to human skin [19]. The skin was prepared as described in earlier publications of our group [20, 21]. Fresh pig ears were cleaned with isotonic saline. Full‐thickness skin was removed from the cartilage, and blood was removed with isotonic saline and cotton swabs. The obtained postauricular skin was dried with soft tissue and sliced into strips of about 3 cm in width. The skin was stretched onto a Styrofoam plate to reduce the impact of wrinkles. With an electric hair trimmer (QC5115/15 Philips Electronics, NL‐Eindhoven, The Netherlands), bristles were cut to about 0.5 mm in length. After being dermatomed to a thickness of 1.0 mm (Dermatom GA 630 Acculan 3 TI Aesculap AG & Co. KG, D‐Tuttlingen, Germany), the skin was punched out into circles of 25 mm diameter and placed in the freezer at minus 28°C wrapped in aluminium foil. On the day of the experiment, the samples were thawed to room temperature on paper tissue soaked with PBS [21].

### Incubation of skin samples in Franz diffusion cells

Franz diffusion cells are a typical type of analytical setup for determining skin absorption ex vivo, and the method described herein has been used extensively by our group in the past, as described in previous publications [9, 10, 20, 21]. Prior articles from our group thoroughly discuss the strategy for incubating skin samples with emulsifiers [10, 22]. Franz diffusion cells (Gauer Glas, D‐Püttlingen, Germany) were filled with 12 mL degassed, prewarmed (32°C) PBS as the receptor fluid. The skin samples were put on top of the acceptor compartments, and the donor compartments were placed on top of the skin. The Franz diffusion cells were placed in a water bath at 32°C (Lauda type Alpha, Lauda Dr. R. Wobser GmbH & Co. KG, D‐Lauda‐Königshofen, Germany). The receptor fluid was continuously stirred at a 500‐rpm rate (Variomag Poly 15, Thermo‐Scientific, Thermo Electron LED GmbH, D‐Langenselbold, Germany). After an expeditious equilibration period of 30 min, the initial TEWL values were generated using the protocol described in 2.7. After the initial TEWL measurements, 1 mL of SLS solution was applied to the respective skin samples to induce skin barrier impairment. Each donor compartment was then covered with a piece of parafilm to reduce evaporation. After a 4‐h incubation, the residual SLS solution was wiped off the skin, and the second TEWL measurement was performed [10, 22]. After that, the formulations or water were applied to the skin samples for another 4 h Formulations were applied as previously validated using a rod made of acrylic glass with around 4 mg for each sample to mimic in‐use conditions as described in Liu et al. [13]. Afterwards, another final TEWL measurement was performed. Experiments were performed in triplicate.

### Measurement of trans‐epidermal water loss (TEWL)

The TEWL was measured by basic device Multi Probe Adapter MPA 6 and probe In‐vitro‐Tewameter® VT 310 (Courage & Khazaka electronic GmbH, D‐Köln, Germany) and calculated by the respective software. Room temperature was 22°C, and relative humidity (RH) was 25% (Klima logg pro TFA 30.3039 IT; Dostmann GmbH & Co. KG, D‐Wertheim, Germany) skin temperature was 32°C. After 30 min, each Franz diffusion cell was taken out of the water bath, and 2 mL PBS was taken out of the Franz diffusion cell acceptor compartment with a needle attached to a 2 mL syringe to facilitate TEWL measurements. After taking off the solution of SLS or the formulation from the donor compartment, the skin was dried with tissues and cotton swabs. The TEWL probe was placed on the acceptor compartment and the extracted 2 mL of PBS were returned back to the Franz diffusion cell. The Franz diffusion cell was placed back into the water bath, and the initial TEWL value was measured. The measurement started with a measurement time of 90 s for each run. A minimum of five measurements were taken and regarded as the equilibration phase. More than five measurements were necessary if the difference between the three subsequent measurements exceeded ±1.00 g·m^−2^·h^−1^. After the last measurement, the probe was removed, and the donor chamber was put back on top of the Franz diffusion cell. At first, 1 mL of SLS was pipetted on the samples, incubated for 4 h to impair skin. Afterwards, SLS solution was discarded, the skin was dried, and the TEWL was measured, as described above. For the second incubation, the respective formulation or water (1 mL) was applied to the skin, and the incubation of 4 h started again. Afterwards, the formulations or water were discarded, the skin was dried, and the TEWL was measured again, as described above. The TEWL change is expressed as the TEWL margin before and after the 4 h of incubation in g·m^−2^·h^−1^. This procedure was validated and is already described in detail in a prior article by our group [23].

### SC isolation and drying

The trypsin digestion process isolated the SC as described by Kligman et al. [9, 24]. This isolation procedure has been proven not to influence the lipid content or the lipid lamellar organization [25]. The obtained skin samples were placed dermal side down on filter paper soaked with 0.2% trypsin diluted in PBS solution. After incubating the skin samples overnight, the SC was peeled off gently and immersed in 0.05% trypsin inhibitor diluted in PBS solution for 1 min. Afterward, the isolated SC was washed with fresh, purified water five times. Before the measurements, samples were stored in a desiccator for drying for 3 days [14].

### Pyranine staining

After trypsin digestion, as described in 2.8., the SC samples were stained with an aqueous pyranine (3 mg/mL) solution to visualize the lipid matrix between the corneocytes. After 60 s of staining, the samples were washed thrice with deionized water for 20 s in a different beaker. The dried samples, stored for a minimum of 3 days in a desiccator, were examined for fluorescence under illumination with blue light (450–490 nm) of a microscope (Microscope Axio Imager Z1, Carl Zeiss AG, D‐Oberkochen, Germany) with a 20× objective (numerical aperture 0.8, Plan‐Apochromat, Carl Zeiss AG, D‐Oberkochen, Germany) and respective software Zeiss Zen Blue (Carl Zeiss AG, D‐Oberkochen, Germany). This procedure was previously described by Zhang et al. [9] and Pagnoni et al. [16].

### Statistical analysis

Three replicates (*n* = 3) were used to originate the data. Kruskal–Wallis test was used with GraphPad Prism 8.0 to detect statistical differences (GraphPad Software Inc., La Jolla, CA, USA). Different numbers of asterisks are used to indicate significant differences: * *p* < 0.05, **p < 0.01, and ***p < 0.001.

## RESULTS

### Formulation and process development

**FIGURE 1:**
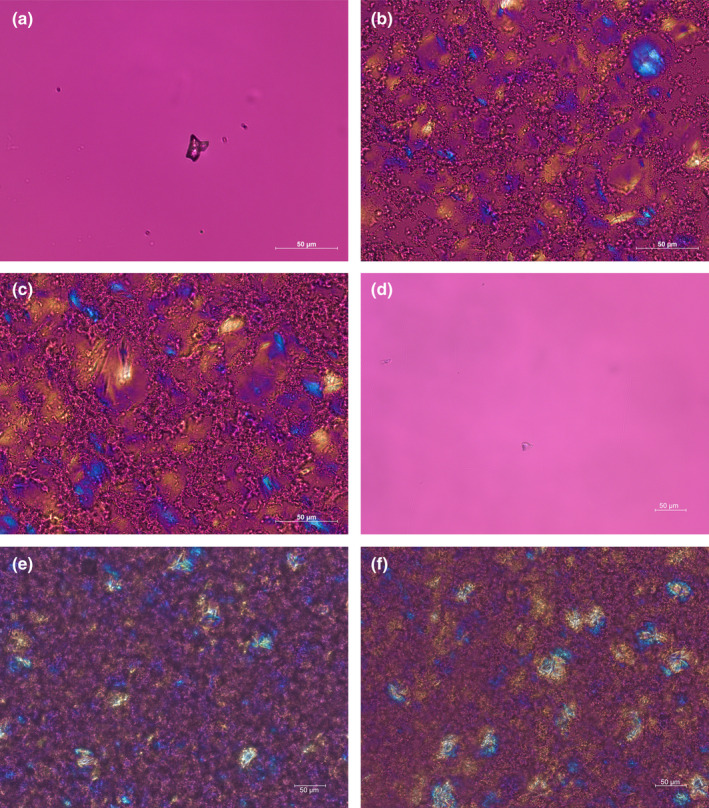
Microscopic images of stability assessment of formulations R1 and R2 with cholesterol in MCT as positive control after 3 months of storage. (a) Shows cholesterol in MCT on the first day. The sample was heated up like the formulations were produced. (b) Shows the formulation R1 immediately after manufacturing. (c) Shows the formulation R2 immediately after manufacturing. (d) Shows cholesterol in MCT after 3 months of storage at room temperature. (e) Shows the formulation R1 after 3 months of storage at room temperature. (f) Shows the formulation R2 after 3 months of storage at room temperature.

During the formulation development, the impact of the parameters of the mixing system on the formulations microstructure were checked by polarization microscopy [13]. The homogenous distribution of white soft paraffin and the absence of cholesterol crystals were aimed for. The homogenous distribution of white soft paraffin was improved by increasing the mixing time from 2 to 4 min and the shear rate from 1000 to 1500 rpm. A higher mixing time of 6 min or a shear rate of 2000 rpm did not improve the formulation further. At the highest shear rate, foaming effects appeared (air was trapped in the formulation) and decreased physical stability, which was macroscopically visible. Thus, 4 min at 1500 rpm were chosen as optimal settings. After 1, 2 and 3 months, the formulations R1 and R2 were checked for cholesterol crystals or any other modifications. Images after storage are slightly darker than after manufacture which may be a result of a slightly thicker sample between slide and cover slip. Altogether, the structure and texture did not change. No cholesterol crystal growth was found as shown in Figure 1a–f. The distribution of white soft paraffin was not influenced either. So we conclude that no relevant changes in microstructure took place during storage.

The different formulations were checked with respect to their macroscopic appearance. The formulation should be a cream with medium viscosity and a white homogeneous colour. White soft paraffin was reduced from 65% to 40% for R1 and 50% to 35% for R2 because, under the polarizing microscope, many areas of white soft paraffin were found not to have been homogenized. This was additionally improved by increasing mixing speed and time, as described above. Accordingly, the amount of water was increased. After colouring the phases R1 and R2 with methyl orange and blue‐violet, both were determined to be oil in water (o/w) creams. As sorbitan monostearate is a weak emulsifiers with low HLB‐value (hydrophilic–lipophilic balance), Polyethylene glycol 100 stearyl ether is a high HLB emulsifier, and the amount of water in the formulation was high, this behaviour was expected.

For the following test on ex vivo porcine skin, the formulations R1 and R2 were selected because they showed the best results in comparison to the self‐determined requirements.

### Formulation characterization by rheology

**FIGURE 2:**
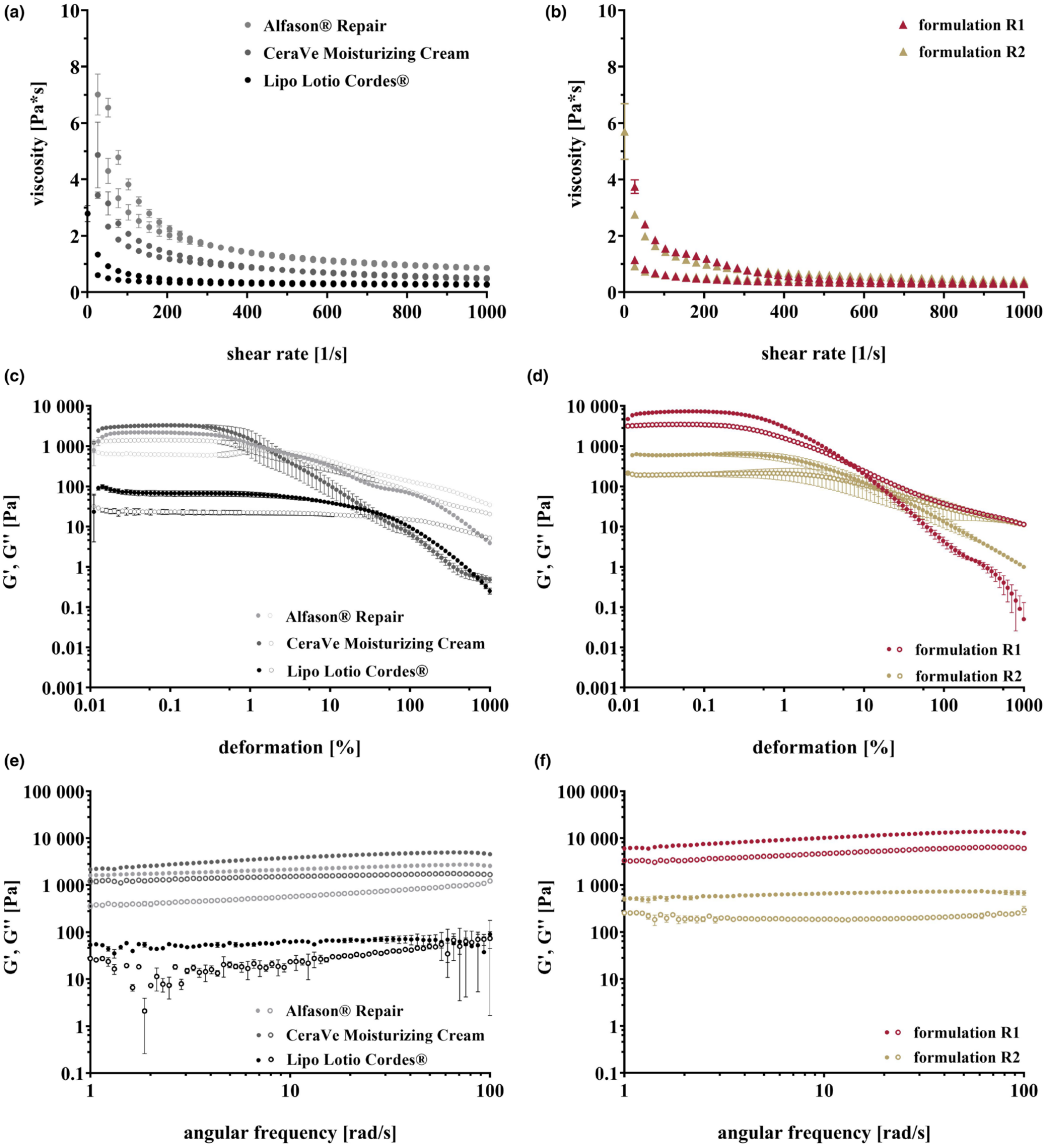
Viscosity curves of formulations (a) and market products (b). Amplitude sweep for formulations (c) and market products (d). Frequency sweep for formulations (e) and market products (f). Mean ± SD, *n* = 3.

The aim was to create a medium viscosity formulation that is easy to apply even on damaged skin. Since the spreadability of semisolid formulations is a consequence of the viscoelastic behaviour, rheology is a suitable and valid method for characterization [26]. The formulations R1 and R2 and three market products with different viscosities were analysed. The results in Figure 2b show that the dynamic viscosities of the formulations R1 and R2 are between one marketed product with low viscosity and two with higher viscosity. The formulations thus appear to show appropriate viscosities. R1 and R2 showed shear thinning behaviour and a small hysteresis area under shear stress. This pseudo‐plasticity is desirable as it improves application through a sliding effect and creates a pleasant feeling [27].

The amplitude and frequency sweeps are shown in Figure 2c–f. Both formulations R1 and R2 show gel‐like behaviour with the storage modulus (G' > G“) being higher than the loss modulus. The formulation R1 shows higher values than R2 due to a stiffer gel network built by the single emulsifier sorbitan monostearate, which is solid at room temperature and forms crystalline networks in the cream. The emulsifier mixture with S100 in R2 reduces the stiffness of the network, as the emulsifier is liquid at room temperature. Thus, the crystalline network is loosened. The crossover indicates the point at which both moduli are equal (G' = G”). Here, the character shifts from an elastic‐dominated behaviour to a viscous‐dominated behaviour and the sample begins to flow. For both developed formulations, the crossover point is between those of the marketed products indicating a structural strength in the same order of magnitude. At high shear rates during application, both formulations will show viscous flow, regardless of the composition. The gel strength (G' in the LVE range) of the developed formulations is similar to or higher than the gel strength of the marketed products. The consistently high values during the frequency sweep (Figure 2e) show a high short‐ and long‐term stability of the inner structure of the formulations [28]. The different emulsifier composition has no influence on the rheological stability. The market products show similar behaviour, although the recognizable fluctuations suggest a lower short‐term stability of the product at higher frequencies. It can be assumed that user acceptance of the developed formulations is high because of the similar rheological properties compared to marketed products.

### Ex vivo characterization: TEWL and pyranine staining

**FIGURE 3:**
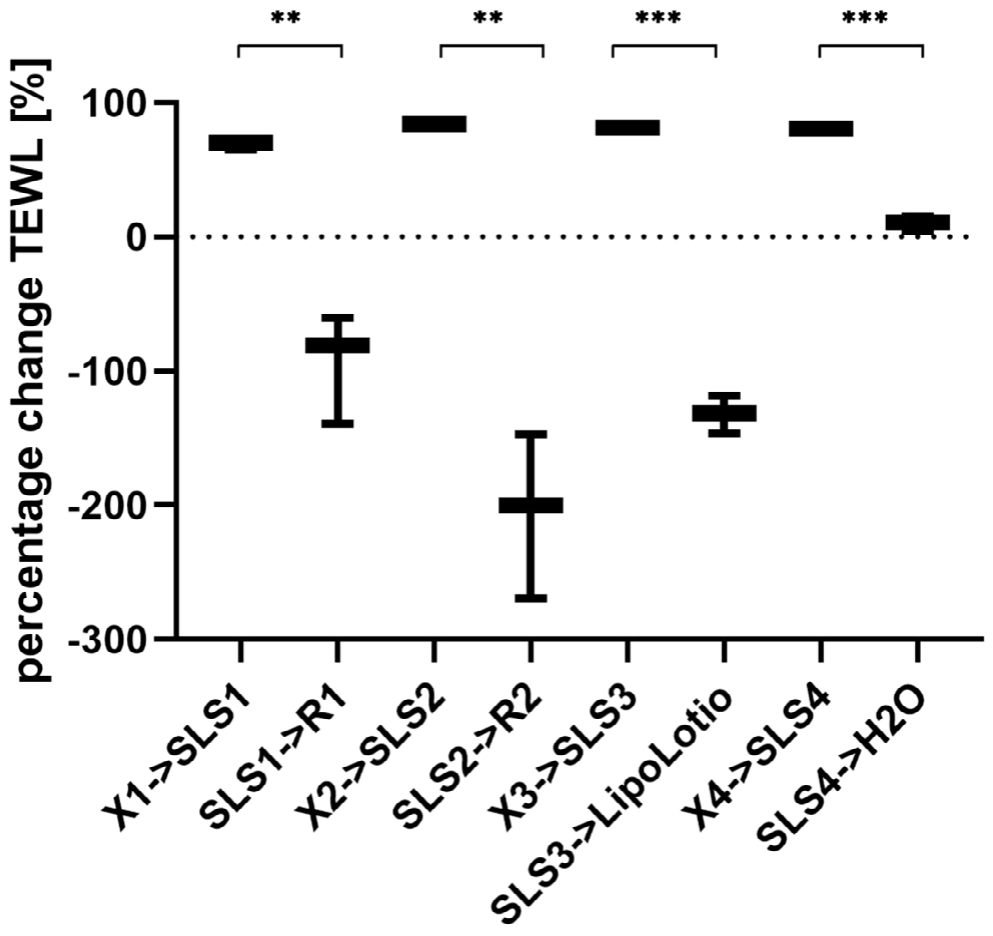
TEWL measurements of skin samples treated with formulations or water as the negative control. X1–4 stands for the samples (*n* = 3) at the initial measurement of four TEWL experiments. X1‐4‐ > SLS1‐4 indicates the difference between the first and the second measurements of the samples exposed to SLS. SLS1‐4‐ > R1/R2/Lipo Lotio Cordes®/H_2_O represents the difference of the second measurements with the third measurements. Results are shown as the change in TEWL percentage. Formulations R1, R2, market product showed significant improving TEWL effects (values in minus scale). Whereas every SLS‐treated sample showed significant adverse effects (values in positive scale). The water control sample showed no change, shown as zero value, after SLS treatment. Mean ± SD, *n* ≥ 3. *p < 0.05, **p < 0.01, ***p < 0.001.

**FIGURE 4:**
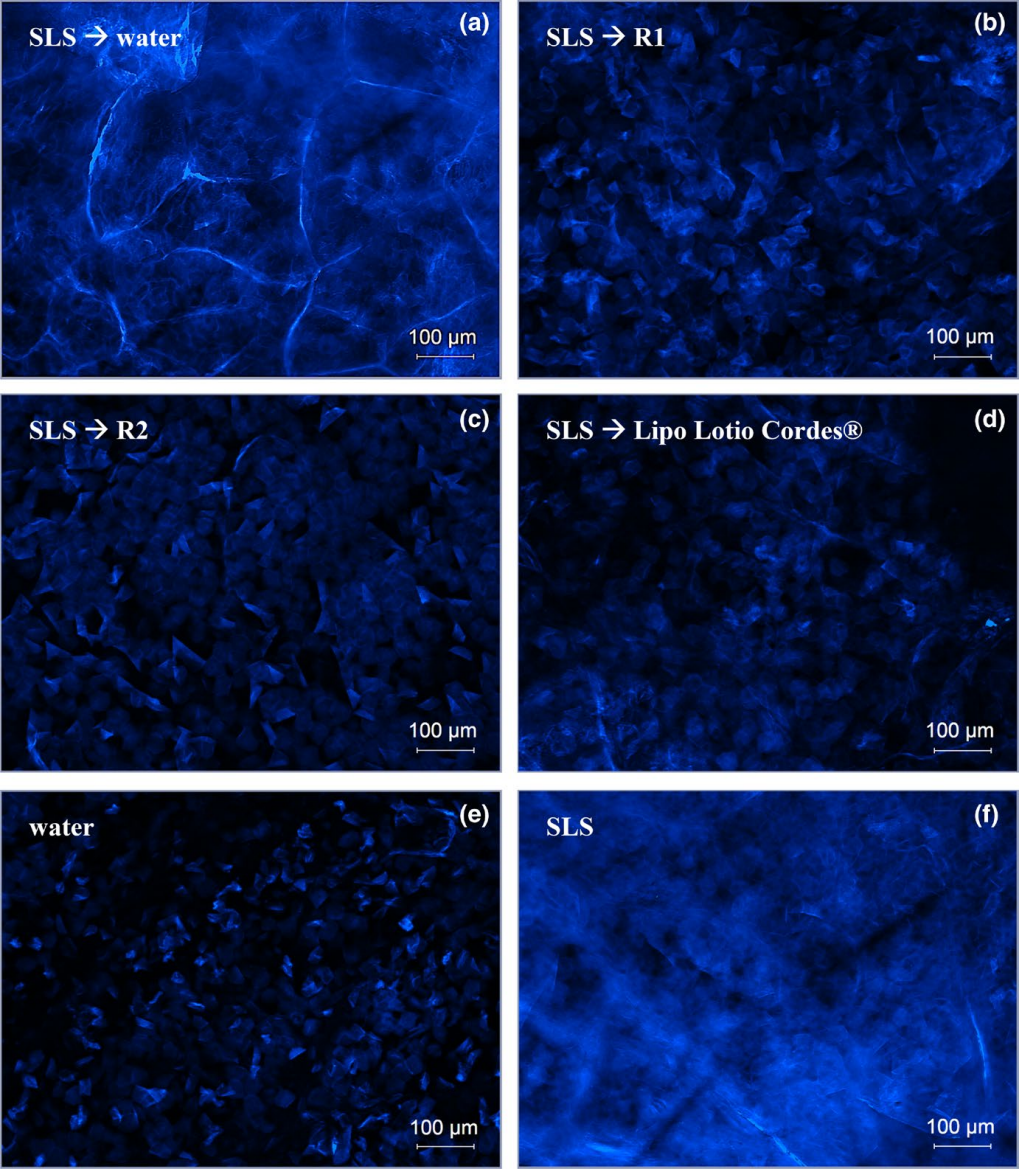
Microscopic images of pyranine stained stratum corneum after treatment of SLS‐impaired skin with water (a), R1 (b), R2 (c) and market product Lipo Lotio Cordes® (d). Skin treatment with water (e) as negative control and SLS as positive control (f) as positive control.

The TEWL values were detected thrice in this study. The first measurement was used to check the skin integrity prior to the experiment. This is also recommended by the EMA draft guideline on the quality and equivalence of topical products [29]. The second measurement was to check if SLS impaired the skin barrier function. The damage, was expected as SLS had been found in our prior research to extract lipids from the skin [4, 5, 6, 7, 8, 9, 10]. To thus impaired skin, the formulations were applied and skin barrier function improvement, which means a reduction of TEWL values, was checked for in the third TEWL measurement. Finally, the TEWL margin was calculated. The results of the TEWL measurements are shown in Figure 3. As expected, SLS, as the positive control, showed significantly increased TEWL values for all samples (on average 80 g·m^−2^·h^−1^). SLS is skin‐disruptive [7] and has shown highly increased TEWL values in previous experiments [23] which can be explained by lipids extraction from the SC [9, 10, 13]. After applying the formulations, the TEWL decreased for the formulations R1, R2, and the marketed product Lipo Lotio Cordes®. The negative control, 1 mL of deionized water, showed no reduction. The formulations and the marketed product improved the skin barrier function. In comparison, R2 showed the strongest improvement, evidenced by the highest extent of TEWL change. We assume that the delivery of lipids to the skin is the main reason for improvement of the skin barrier. Consequently, the skin can hold back water, and the TEWL values decrease. In contrast, water did not stabilize the skin barrier function, and increased TEWL values persisted. This is in accordance with our previous research, where formulations showed positive effects on the skin barrier function [9]. Pyranine staining was used to visualize the lipids in the SC and to test our assumption of the formulations replacing previously extracted lipids as a reason for skin barrier improvement. The method is based on the binding capacity of keratin [9, 16]. The more keratin filaments are exposed in the SC due to an impairment of the skin barrier as a result of lipid extraction, the more intense the binding of pyranine to keratin and, thus, the more intense the colour. Lipid extraction was performed by incubating the skin with SLS. The damaged but subsequently untreated skin served as a positive control, while undamaged skin incubated only with water served as a negative control. The developed formulations, the marketed products or water were applied to the impaired skin. All samples were subsequently stained with pyranine to draw conclusions regarding the intensity of the lipid extraction. The references are important for interpreting the skin barrier impairment of formulations or ingredients. Because the results are a visual analysis of brightness or darkness, water, as a negative control, has no influence on the SC lipids and shows dark colour, while SLS, as a positive control, extracts lipids and thus give a bright colour. As expected, there was a clear difference between these two control samples visible in Figure 4: the water‐treated sample (Figure 4e) had fewer bright areas in a dark blue background, whereas the SLS‐treated sample (Figure 4f) showed high brightness and a little dark background. This shows that, as expected, the SLS‐treated sample exhibits much more skin barrier damage than the water‐treated one. An intense coloration, comparable to the positive control, can also be seen when the skin was treated with water (Figure 4a) after exposure to SLS. The treatment with the formulations R1 (Figure 4b), R2 (Figure 4c), and the marketed product Lipo Lotio Cordes® (Figure 4d) shows only a slight coloration which is comparable to the negative control (water‐treated sample). The formulation R2 lead to increased recovery compared to the marketed product and R1 which is shown by the different colour intensity. Therefore, lipid replenishment is possible by treatment with the formulations. A difference in the degree of skin barrier restoration was observed after treatment with different formulations. Therefore, not every formulation is equally suitable for the treatment of damaged skin and in our case, R2 outperformed the other formulations.

## DISCUSSION

Treating chronically diseased skin is challenging and depends to a large extent on the patient's compliance. Lifelong basic therapy is necessary [1]. The design of creams or ointments, therefore, has to follow the customers' wishes, like white colour and smooth touch on their skin or quick absorption without stickiness. During formulation development, objective measures like microscopy and rheology are preferred over subjective perception and were thus also used in the current study. The results showed that the creams had a well‐distributed water and oil phase, with no particles like cholesterol crystals or other agglomerates. The rheological properties showed a cream of medium viscosity with good short‐ and long‐term stability. To test the effect of the formulation on impaired skin, porcine skin samples were first partially extracted with SLS and afterwards treated with the developed creams and the marketed reference product. The well‐established method of TEWL measurements was used to detect the different effects on the skin barrier function. The creams showed positive effects by decreasing TEWL values after barrier impairment. The precision of the TEWL measurement has already been shown, and TEWL is a suitable tool to characterize the impact of formulations on the skin barrier function [23, 30, 31]. As the samples were thoroughly cleaned from the cream, no occlusion effects or residual amounts of the formulations could have influenced the TEWL measurement. The decrease in TEWL values after 4 h of incubation with the formulations showed the ability to improve the skin barrier function as an important part of basic therapy. In addition to the TEWL values, pyranine staining followed by fluorescent microscopic analysis is another well‐established method to detect skin barrier impairment visually after exposure to creams or other semisolid or liquid skin care products [9]. The samples treated with creams showed repaired skin with fewer bright areas, similar to the negative control. The difference to the positive control with much more brightness was visible. Therefore, lower accessibility to keratin filaments and, thus, an increased skin barrier function as a result of higher lipids content can be deduced. Combining the results of these two methods, it is obvious that the developed creams, especially R2, positively affected the SC as compensation for the initial damage caused by SLS was achieved after the formulations were applied. The use of sorbitan esters, the most common w/o‐emulsifiers in pharmaceutical formulations [32, 33, 34], can thus be recommended for formulations for basic therapeutics.

## CONCLUSION

Basic therapy is an essential pillar of therapy for chronically diseased skin. Patients mostly use different cosmetic or pharmaceutical products for the prevention or treatment of chronic disease manifestations of the skin. Products are usually selected without sufficient basic knowledge of the ingredients contained in the preparations and their suitability for damaged skin. This is cost‐intensive, and most cosmetic products are not well‐analysed with respect to the adverse effects of ingredients. Even products with SLS are still commonly used although the negative effect of this substance has already been extensively studied. The testing of new and old formulations for suitability on diseased skin should be focused on in the future to ensure that no damage occurs to patients. Formulations have been developed that can be produced cost‐effectively on site using standard manufacturing methods e.g., in hospital pharmacies with the function of increasing the integrity of damaged skin acutely or preventively. They are therefore attractive alternatives to market products and can be of great benefit in the basic therapy of chronic skin diseases.

In the next steps, the formulations R1 and R2 should be tested in human patients with chronic skin diseases to gain access to a product for basic therapy that can be produced in local and clinical pharmacies.

## ACKNOWLEDGMENTS

Open Access funding enabled and organized by Projekt DEAL.

## CONFLICT OF INTEREST STATEMENT

The authors declare no conflicts of interest.

## ETHICS STATEMENT

Porcine ears were obtained from a local butcher after the animal's death. Before the study, the Department of Pharmaceutical Technology was registered with the District Office of Tuebingen to utilize animal products (registration number: DE 08416105221).
